# Multiple parasitic crustacean infestation on belonid fish *Strongylura strongylura*

**DOI:** 10.3897/zookeys.457.6817

**Published:** 2014-11-25

**Authors:** Panakkool-Thamban Aneesh, Kappalli Sudha, Ameri Kottarathil Helna, Gopinathan Anilkumar, Jean-Paul Trilles

**Affiliations:** 1 Post Graduate Department of Zoology and Research Centre, Sree Narayana College, Kannur 670 007 India; 2 School of Biosciences and Technology, VIT University, Vellore 632014 India; 3 UMR 5119 (CNRS-UM2-IRD-UM1-IFREMER), Equipe Adaptation Ecophysiologique et Ontogenèse, University of Montpellier 2, CC. 092, 34095 Montpellier Cedex 05, France

**Keywords:** Isopod, Copepod, quadruple parasitism, triple parasitism, double parasitism, *Strongylura strongylura*

## Abstract

Simultaneous multiple infestation of parasitic crustacean species involving a cymothoid isopod, *Cymothoa frontalis* Milne Edward, 1840 and four species of copepods such as *Lernanthropus tylosuri* Richiardi, 1880, *Caligodes lacinatus* Kroyer, 1863, *Bomolochus bellones* Burmeister, 1833 and *Dermoergasilus coleus* Cressey & Collette, 1970 was frequently noticed on spot-tail needlefish, *Strongylura strongylura* (Belonidae) captured from the Malabar coast (Kerala, India) during the period from April 2011 to March 2012. All the 43 fishes (*Strongylura strongylura*) collected, were under the hyper-infection with parasitic crustaceans; a total of 388 parasitic crustaceans including 57 *Cymothoa frontalis*, 252 *Lernanthropus tylosuri*, 31 *Caligodes lacinatus*, 24 *Bomolochus bellones* and 32 *Dermoergasilus coleus* were recovered from the host fish. 4 members (9.30%) of host fish were under quadruple parasitism, in two different combinations. Seventeen (39.53%) host fishes showed triple parasitism and 20 (46.51%) members exhibited double parasitism, with four and five parasitic combinations respectively. Remaining two (4.65%) fishes were parasitized only by the copepod, *Lernanthropus tylosuri*. The infestations by all recovered parasitic crustaceans were highly site specific. The damage caused by the parasitic crustaceans was also discussed.

## Introduction

Parasitic diseases in fish seriously limit aquaculture production and its economic viability; knowledge of fish parasites, therefore, is an essential requirement for successful aquaculture (Elshahawy and Desouky 2012). Parasitic crustaceans infesting the fishes generates considerable parasitological interest and is of great economic importance inasmuch as it could adversely affect the health of food fishes (Karlsbakk et al. 2001, Johnson et al. 2004, Trilles et al. 2011, 2012, Aneesh et al. 2013a). They feed on the host mucus, tissues and blood and inflict fatal injuries through secondary infection (Margolis et al. 1975, Margolis and Kabata 1988; Oktener and Sezgin 2000). With the increased development of semi-intensive and intensive, freshwater, brackish water and marine aquaculture, the importance of the study of parasitic crustaceans as a major pest has become more evident inviting the attention of many investigators throughout the world (Johnson et al. 1996, 2004, Williams and Williams 1998; Izawa and Choi 2000, Ho et al. 2000, Karlsbakk et al. 2001, Hadfield et al. 2010, 2011, 2013).

Most of the parasitic crustaceans belong to Isopoda, Branchiura and Copepoda (Margolis et al. 1975, Oktener and Sezgin 2000). Cymothoids are oligoxenous isopods and often induce deleterious effects on the host (Overstreet 1978, Kabata 1985, Trilles and Hipeau-Jacquotte 1996, 2012, Aneesh et al. 2013a). Significant proportion of the parasitic copepods is known to be parasitizing fishes (Kabata 1979, Love and Moser 1983, Hogans and Dadswell 1985, Pillai 1985, Benz 1986, Oldewage and Smale 1993, Benz et al. 2003, Cheng et al. 2009, 2011, Ho et al. 2010, El-Rashidy and Boxshall 2010, 2012). Indian fishes have been shown to possess high rate of susceptibility for parasitization by isopods and copepods as well (Pillai 1985, Aneesh et al. 2012, 2013b; Trilles et al. 2011, 2012, Helna et al. 2013, Vijayakumar et al. 2013, Bharadhirajan et al. 2013).

Reports are scanty on the simultaneous occurrence of multiple parasitism involving exclusively parasitic crustaceans. Daniel and Rao (1967) and Daniel and Premkumar (1967) reported the simultaneous infestation of flying fish (*Cypselurus speculiger*) by a copepod, *Pennella* sp. and the cirriped, *Conchoderma virgatum*. Hewitt (1979) and Benz et al. (2003) observed the multiple infestation of Pacific white shark (*Carcharodon carcharias*) by 5–8 different siphonostomatoid (copepod) species. In India, incidence of double parasitism involving the isopod, *Nerocila phaiopleura* and the copepod, *Lernaeenicus sprattae* was reported in anchovy fish, *Stolephorus commersonii* (Rajkumar et al. 2006). Another Indian fish (*Hemiramphus far*) also showed simultaneous infestation by the isopod, *Mothocya plagulophora* and the copepod, *Lernaeenicus hemiramphi* (Gopalakrishnan et al. 2010). The simultaneous multiple infestations by four parasitic crustacean species on banded needle fish, *Strongylura leiura* was recently reported by Aneesh et al. (2013b).

The present study reports the frequent occurrence of double and triple parasitism and also the few incidence of quadruple parasitism exclusively by the species of parasitic crustaceans including isopod and copepods on the host fish, *Strongylura strongylura* (Belonidae) distributed along the Malabar coast (Kerala, India).

## Methods

The present study was conducted during the period from April 2011 to March 2012. Living or fresh fishes, *Strongylura strongylura* were collected from the Ayyikkara fish landing center (Lat. 11°51'N, Long. 75°22'E, Malabar coast, Kerala, India). Soon after collection, the fishes were taken to the laboratory and were examined various parts of the body (such as the general body surface, the lateral line region, base of the pectoral fin, posterio-ventral side, branchial cavity, beak, gill filament, inner wall of the operculum etc.) thoroughly for the presence of parasitic crustaceans using hand lens. Recovered parasitic crustaceans were removed from the host and preserved in 70% ethanol for further detailed examination. The identification was performed, using a dissection microscope and a stereo microscope Leica-S6D, according to Milne Edwards (1840), Cressey and Collette (1970) and Pillai (1985). The prevalence (P) and mean intensity (I) was calculated according to Margolis et al. (1982) and Bush et al. (1997). The host nomenclature and fish taxonomy were done according to Fish Base (Froese and Pauly 2013).

Voucher specimens of all parasites, collected by Aneesh, Helna and Sudha, from the fish, *Strongylura strongylura*, were deposited in the Parasitic Crustacean Museum, Crustacean Biology Research Laboratory, Sree Narayana College, Kannur, Kerala, India. Abbreviations used: PCM – Parasitic Crustacean Museum, Crustacean Biology Research Laboratory, Sree Narayana College, Kannur, Kerala, India; LT – Total length.

*Cymothoa frontalis* (Milne Edward, 1840): Juvenile (LT. 8 mm) (PCM N° CF-07),13 April 2011; Transitional (LT. 20mm) (PCM N° CF-08), 25 April 2011; ovigerous female (LT. 26 mm) with manca larva in the brood pouch (PCM N° CF-09 ), 12 July 2011; 30 Manca larva released by the specimen PCM N° CF-09 (3.4 mm), (PCM N° CF-10), 12 July 2011; Male (LT. 13 mm) (PCM N° CF-14), 08 January 2012.

*Caligodes lacinatus* (Kroyer, 1863): Ovigerous female (LT, 10.3 mm) with egg sac (PCM N° *Cl*-09), 07 May 2011; ovigerous female (LT, 6 mm) without egg sac (PCM N° *Cl*-13), 23 July 2011.

*Lernanthropus tylosuri* Richiardi, 1880: Ovigerous female (LT, 5.5 mm) with egg sac (PCM N° *Lt*-01), 07 May 2011; ovigerous female (LT, 5.5 mm) without egg sac and a male (LT, 1.8 mm) clinging on Ovigerous female (PCM N° *Lt*-13), 23 March 2012.

*Bomolochus bellones* Burmeister, 1833: Ovigerous female (LT, 1.9 mm) with egg sac (PCM N° *Bb*-09), 18 June 2011; ovigerous female (LT, 1.8 mm) with egg sac (PCM N° *Bb*-11), 23 June 2011; ovigerous female (LT, 1.4 mm) without egg sac (PCM N° *Bb*-12), 23 June 2011.

*Dermoergasilus coleus* (Cressey & Collette, 1970): Ovigerous female (LT, 0.6 mm) with egg sac (PCM N° *Dc*-05), 19 July 2011; ovigerous female (LT, 0.5 mm) without egg sac (PCM N° *Dc*-12), 19 January 2012.

## Results

**Figure 1.**
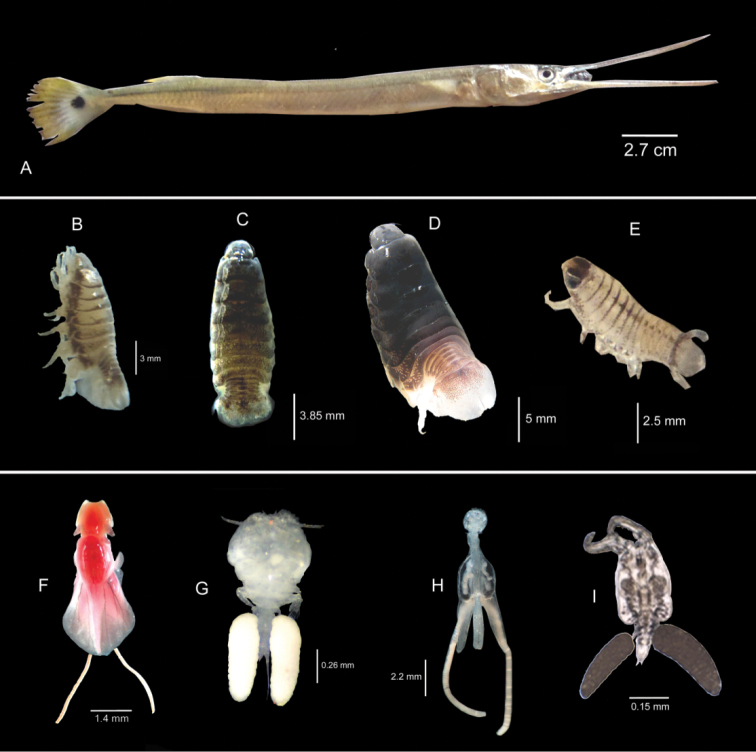
**A** Host fish *Strongylura strongylura* **B–E** *Cymothoa frontalis* **B** male **C** transitional **D** female **E** juvenile **F** *Lernanthropus tylosuri* – female **G** *Bomolochus bellones* – female **H** *Caligodes lacinatus* – female **I** *Dermoergasilus coleus*.

Forty three *Strongylura strongylura* (Fig. 1A) collected during April 2011 to March 2012, were found to be infested with five species of parasitic crustaceans. Recovered parasitic crustaceans were identified as cymothoid isopod, *Cymothoa frontalis* (Milne Edward, 1840) and four copepods (such as *Lernanthropus tylosuri* Richiardi, 1880, *Caligodes lacinatus* Kroyer, 1863, *Bomolochus bellones* Burmeister, 1833 and *Dermoergasilus coleus* (Cressey & Collette, 1970) (Tables 1, 2 and 3) (Figs 1B–I).

**Table 1.** Multiple parasitism by crustaceans on *Strongylura strongylura*.

Month	Host fish series	Number of Parasitic Crustaceans										Remarks
		Isopod		Copepods								
		*Cymothoa frontalis*		*Lernanthropus tylosuri*		*Caligodes lacinatus*		*Bomolochus bellones*		*Dermoergasilus coleus*		
		Female	Male	Female	Male	Female	Male	Female	Male	Female	Male	
April 2011	1	1	1	12	3	1	-	-	-	-	-	Triple
	2	1	1	7	1	-	-	2	-	-	-	Triple
	3	1	1	-	-	-	-	1	-	-	-	Double
	4	1	-	4	1	-	-	-	-	-	-	Double
	5	-	-	4	-	2	-	-	-	-	-	Double
May 2011	6	-	-	8	-	-	-	1	-	-	-	Double
	7	1	-	4	-	-	-	-	-	-	-	Double
	8	1**	1**	-	-	2	-	-	-	-	-	Double
June 2011	9	1	1	11	2	2	-	-	-	-	-	Triple
	10	-	-	4	-	-	-	-	-	-	-	Single
	11	1	1	-	-	-	-	2	-	-	-	Double
	12	1	-	3	-	-	-	-	-	-	-	Double
July 2011	13	1**	1**	9	9	2	-	-	-	-	-	Triple
	14	1	1	-	-	-	-	1	-	-	-	Double
	15	-	-	3	3	-	-	-	-	-	-	Single
Aug 2011	16	1*	1	-	-	2	-	2	-	4	-	Quadruple
	17	1	1	5	1	-	-	-	-	-	-	Double
	18	-	-	10	-	1	-	-	-	-	-	Double
	19	1	-	8	-	-		1		4		Quadruple
	20	-	-	6	1	2	-	-	-	-	-	Double
	21	1	1	4	-	-	-	-	-	-	-	Double
September 2011	22	1	1	9	2	-	-	2	-	-	-	Triple
	23	1*	1	3	-	2	-	-	-	-	-	Triple
October 2011	24	-	-	9	-	1	-	-	-	3	-	Triple
	25	1	1	7	-	-	-	-	-	3	-	Triple
	26	1	1	2	1	-	-	1	-	-	-	Triple
November 2011	27	-	-	11	2	2	-	-	-	-	-	Double
	28	1	-	2	-	-	-	2	-	-	-	Triple
	29	1	1	6	-	-	-	-	-	-	-	Double
December 2011	30	1	1	7	-	2	-	-	-	-	-	Triple
	31	1	-	3	-	-	-	2	-	-	-	Triple
	32	1**	1**	8	2	2	-	-	-	-	-	Triple
	33	1	1	-	-	2	-	1	-	3	-	Quadruple
January 2012	34	1	1	-	-	2	-	-	-	-	-	Double
	35	1*	-	7	2	-	-	-	-	3	-	Triple
	36	-	-	5	-	-	-	2	-	-	-	Double
February 2012	37	-	-	4	-	2	-	-	-	-	-	Double
	38	1	1	12	4	-	-	-	-	2	-	Triple
	39	1*	1	-	-	-	-	-	2	-	-	Double
	40	1	1	3	-	-	-	-	-	4	-	Triple
March 2012	41	-	-	9	-	2	-	-	-	-	-	Double
	42	1**	1**	5	-	-	-	2	-	4	-	Quadruple
	43	1	1	4	-	-	-	-	-	2	-	Triple
	43	24 ** - 4 * - 4	21 ** - 4	218	34	29	0	23	0	29	0	Single – **2** Double – **20** Triple – **17** Multiple – **4**
		32(57) * – Transitional stage = 4 ** – Juvenile = 8		35 (252)		17(31)		15(24)		10(32)		

**Table 2.** Parasitological index of the parasitic crustaceans on *Strongylura strongylura* under multiple parasitism.

Parasites	Prevalence and Intensity	Site of infestation
***Cymothoa frontalis*** Milne Edward, 1840	74.42; 1.78	floor of the buccal cavity
***Lernanthropus tylosuri*** Richard, 1880	81.4; 7.2	on the gill filament
***Caligodes lacinatus*** Kroyer, 1863	39.53; 1.82	penetrating the fleshy part of the lower beak
***Bomolochus bellones*** Burmeister 1835	34.88; 1.6	attached on the inner side of the operculum
***Dermoergasilus coleus*** (Cressey in Cressey & Collette, 1970)	23.26; 3.2	on the gill filament

**Table 3.** Different parasitic combinations of multiple parasitism.

NFO	Single Parasitism	Double Parasitism					Triple Parasitism				Quadruple Parasitism	
	L	CL	CCl	CB	LCl	LB	CLCl	CLB	CLD	LClD	CLBD	CClBD
43	2	6	3	3	5	3	6	5	5	1	2	2
		Total – 20					Total – 17				Total – 4	
	Total: 43											

(Legends: CL – *Cymothoa frontalis* and *Lernanthropus tylosuri*; CC*l* – *Cymothoa frontalis* and *Caligodes lacinatus*; CB – *Cymothoa frontalis* and *Bomolochus bellones*; LC*l* – *Lernanthropus tylosuri* and *Caligodes lacinatus*; LB – *Lernanthropus tylosuri* and *Bomolochus bellones*; CLC*l* – *Cymothoa frontalis*, *Lernanthropus tylosuri* and *Caligodes lacinatus*; CLB – *Cymothoa frontalis*, *Lernanthropus tylosuri* and *Bomolochus bellones*; CLD – *Cymothoa frontalis*, *Lernanthropus tylosuri* and *Dermoergasilus coleus*; LC*l*D – *Lernanthropus tylosuri*, *Caligodes lacinatus* and *Dermoergasilus coleus*; CLBD – *Cymothoa frontalis*, *Lernanthropus tylosuri*, *Bomolochus bellones* and *Dermoergasilus coleus*; CC*l*BD – *Cymothoa frontalis*, *Caligodes lacinatus*, *Bomolochus bellones* and *Dermoergasilus coleus*)

**Figure 2.**
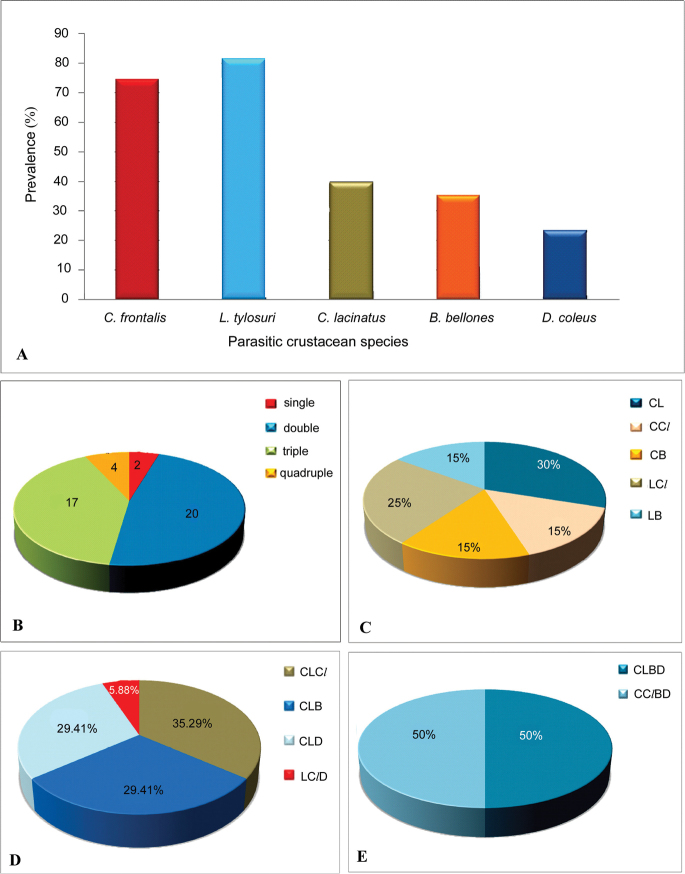
**A** Simultaneous occurrence of parasitic crustaceans (*Cymothoa frontalis*, *Lernanthropus tylosuri*, *Caligodes lacinatus* and *Bomolochus bellones*, *Dermoergasilus coleus*) parasitizing the fish *Strongylura strongylura* **B** Levels of single, double, triple and quadruple crustacean parasitism on the fish *Strongylura strongylura* **C** Double parasitism on the fish *Strongylura strongylura* – different combinations is represented in percentage **D** Triple parasitism on the fish *Strongylura strongylura* – different combinations is represented in percentage **E** Quadruple parasitism on the fish *Strongylura strongylura* – different combinations is represented in percentage. Legends: CL – *Cymothoa frontalis* and *Lernanthropus tylosuri*; CC*l* – *Cymothoa frontalis* and *Caligodes lacinatus*; CB – *Cymothoa frontalis* and *Bomolochus bellones*; LC*l* – *Lernanthropus tylosuri* and *Caligodes lacinatus*; LB – *Lernanthropus tylosuri* and *Bomolochus bellones*; CLC*l* – *Cymothoa frontalis*, *Lernanthropus tylosuri* and *Caligodes lacinatus*; CLB – *Cymothoa frontalis*, *Lernanthropus tylosuri* and *Bomolochus bellones*; CLD – *Cymothoa frontalis*, *Lernanthropus tylosuri* and *Dermoergasilus coleus*; LC*l*D – *Lernanthropus tylosuri*, *Caligodes lacinatus* and *Dermoergasilus coleus*; CLBD – *Cymothoa frontalis*, *Lernanthropus tylosuri*, *Bomolochus bellones* and *Dermoergasilus coleus*; CC*l*BD – *Cymothoa frontalis*, *Caligodes lacinatus*, *Bomolochus bellones* and *Dermoergasilus coleus*.

Among the five parasitic crustaceans recovered from *Strongylura strongylura*, the *Lernanthropus tylosuri* exhibited highest prevalence (81.4%); out of 43 fish (*Strongylura strongylura*) observed 35 member were found to be infested with this lernanthropid copepod (Figs 1F and 2A). A total of 252 (218 females and 34 males) members of *Lernanthropus tylosuri* were recovered from 35 infested fishes and the intensity being 7.2 (Table 2). All females were reproductively active, evidenced by the presence of growing ovaries and/or egg sacs. The recovered males were not independent, but found to be in a clinging/copulatory position, attaching the genital segment of the females with their maxilliped. *Lernanthropus tylosuri* shows strict site specificity by infesting only the gill filament of the host.

Among the recovered parasitic crustacean species, the isopod *Cymothoa frontalis* exhibited the second highest prevalence (74.42%) (Table 2; Figs 1B–E and 2A) by infesting 32 (out of 43) host fishes (*Strongylura strongylura*); the intensity being equal to 1.78 (Table 2). Of 57 members of *Cymothoa frontalis*, there were 21 males and 24 females, 8 juveniles and the remaining 4 were under the transitional phase. Fifty members of this cymothoid parasite were appeared in pairs with three different combinations during their infestation on the fish (*Strongylura strongylura*) such as male–female (18+18=36), juvenile–juvenile (4+4=8) and male–transitional stage (3+3=6); invariably, member in the pair being settled at the floor of either of the branchial cavity of the host fish. The remaining unpaired 7 (57–50) members of *Cymothoa frontalis*, including 6 females and one transitional stage were also found to be settled the specific site (floor of the host buccal cavity).

The copepod species, *Caligodes lacinatus* was collected from 17 out of 43 *Strongylura strongylura* examined; the prevalence being 39.53%. A total of 31 parasites were recovered from the infested host fishes, the intensity being equal to 1.82 (Table 2; Figs 1H and 2A). All the recovered members of this parasite were exclusively females carrying growing ovaries and egg sacs as well. *Caligodes lacinatus* was found to penetrate the fleshy part of the lower beak of the host fish; swelling and tissue damages were frequently observed at the penetration site.

Fifteen of 43 *Strongylura strongylura* examined were also infested by 24 members of the copepod *Bomolochus bellones*. The prevalence and intensity reach 34.88% and 1.6 respectively (Table 2; Figs 1G and 2A). All specimens were females and reproductively active by possessing egg sacs or maturing ovaries. The inner side of the operculum forms the specific site for the attachment of this species.

*Dermoergasilus coleus* showed the lowest prevalence (23.26%), only 10 of the 43 *Strongylura strongylura* examined being infested; 32 parasites were recovered from the gill filament of infested host fishes, the intensity being equal to 3.2 (Table 2; Figs 1I and 2A). All females were reproductively active possessing growing ovaries and/or egg sacs. *Dermoergasilus coleus* also showed strict site specificity by infesting only the gill filament of the fish. The infestation of *Dermoergasilus coleus* is found to be restricted to certain months (August, October and December–March) only.

Interestingly, the host fish (*Strongylura strongylura*) was under frequent and simultaneous multi infestation (quadruple/triple/double) by any four/three/two of these five parasitic crustaceans (*Cymothoa frontalis*, *Lernanthropus tylosuri*, *Caligodes lacinatus*, *Bomolochus bellones* and *Dermoergasilus coleus*) throughout the study period (April 2011 to March 2012) (Table 1; Fig. 2B–E).

### Quadruple parasitism

Approximately 9% of the observed (4 out of 43) fishes showed the presence of quadruple parasitism, being simultaneously infested by any of the four species of parasitic crustaceans in two different combinations (1. *Cymothoa frontalis*, *Lernanthropus tylosuri*, *Bomolochus bellones* and *Dermoergasilus coleus* (CLBD) (50%; 2 out of 4) and 2. *Cymothoa frontalis*, *Caligodes lacinatus*, *Bomolochus bellones* and *Dermoergasilus coleus* (CC*l*BD) (50%; 2 out of 4)) only during the months of August, December and March (Tables 1 and 3; Fig. 2B and E).

### Triple parasitism

Seventeen (out of 43; 39.53%) members of *Strongylura strongylura* showed triple parasitism by simultaneous infestation by any of the three parasitic crustacean species in following four possible combinations: 1) *Cymothoa frontalis*, *Lernanthropus tylosuri* and *Caligodes lacinatus* (CLC*l*), 2) *Cymothoa frontalis*, *Lernanthropus tylosuri* and *Bomolochus bellones* (CLB), 3) *Cymothoa frontalis*, *Lernanthropus tylosuri* and *Dermoergasilus coleus* (CLD) and 4) *Lernanthropus tylosuri*, *Caligodes lacinatus* and *Dermoergasilus coleus* (LC*l*D). The rates of these combinations were 35.29% (CLC*l*), 29.41% (CLB), 29.41% (CLD) and 5.88% (LC*l*D) respectively (Tables 1 and 3; Fig. 2B and D). Instances of triple parasitism were observed throughout the study period except May and August (Table 1).

### Double parasitism

The instance of double parasitism in *Strongylura strongylura* was relatively high. Twenty (out of 43; 46.51%) members of the host fish were under simultaneous infestation with any of the two crustacean species. Five possible combinations of double parasitism were detected 1) *Cymothoa frontalis* and *Lernanthropus tylosuri* (CL), 2) *Cymothoa frontalis* and *Caligodes lacinatus* (CC*l*) 3) *Cymothoa frontalis* and *Bomolochus bellones* (CB) 4) *Lernanthropus tylosuri* and *Caligodes lacinatus* (LC*l*), 5) *Lernanthropus tylosuri* and *Bomolochus bellones* (LB). CL and LC*l* combinations were found to be significantly high amounting 30% and 25% respectively. The percentage of CC*l*, CB and LB combinations were found to be equal (15% each) (Table 3; Figs 2B and C). No incidence of double parasitism was noticed in September, October and December (Table 1).

### Single parasitism

Unlike triple and double parasitism noticed in the studied host fish *Strongylura strongylura*, infestation with only one species of parasitic crustacean (single parasitism) was uncommon during the entire study period; only two fishes (out of 43; 4.65%) showed single parasitism with *Lernanthropus tylosuri*, one in June and other in July (Tables 1 and 3; Fig. 2B).

## Discussion

The present study revealed that the spot tail needle fish, *Strongylura strongylura* is a potential host for accommodating five parasitic crustacean species. 396 parasitic crustaceans including 57 cymothoid isopod (*Cymothoa frontalis*) and 339 copepods (252 *Lernanthropus tylosuri*, 31 *Caligodes lacinatus*, 24 *Bomolochus bellones* and 32 *Dermoergasilus coleus*) were recovered from 43 examined fish, *Strongylura strongylura*. The highest prevalence (P = 81.4%) was exhibited by the copepod, *Lernanthropus tylosuri* throughout the study period. The parasitic cymothoid, *Cymothoa frontalis* was recovered from 32 (P = 74.42%) host fishes (*Strongylura strongylura*). The parasitic copepod, *Caligodes lacinatus* was collected from 17 host fishes, its prevalence being 39.53%. The prevalence of *Bomolochus bellones*, infesting only 15 of 43 examined fishes, being 34.88 %. *Dermoergasilus coleus* parasitizing 10 *Strongylura strongylura* exhibited lowest prevalence (23.26%). The mean intensity vary according to the parasitic species. The highest intensity was observed in *Lernanthropus tylosuri* (I = 7.2), the second highest intensity was exhibited by *Dermoergasilus coleus* (I = 3.2). *Caligodes lacinatus* and *Cymothoa frontalis* have an intensity reaching 1.82 and 1.78 respectively. The lowest intensity was observed in *Bomolochus bellones* (1.6). Among the four copepod species recovered during the present study, the species such as *Lernanthropus tylosuri*, *Caligodes lacinatus* and *Bomolochus bellones* were also reported to be the members in simultaneous multiple infestation on *Strongylura leiura* (Aneesh et al. 2013b). But the intensity and prevalence of *Lernanthropus tylosuri* is found to be higher in *Strongylura strongylura* than *Strongylura leiura*, suggesting that *Strongylura strongylura* is a more suitable host fish for *Lernanthropus tylosuri*. The prevalence of *Caligodes lacinatus*, on the other hand, found to be very low in the present host (*Strongylura strongylura*). The prevalence and intensity of *Bomolochus bellones* was found to be more or less equal in both fishes, *Strongylura strongylura* (present study) and *Strongylura leiura* (Aneesh et al. 2013b).

Interestingly, these five parasitic crustaceans showed site specific attachment, apparently for avoiding the inter-parasitic competition for space and food. The blood feeding parasite, *Cymothoa frontalis*, prefers floor of the buccal cavity and the copepods, *Lernanthropus tylosuri* and *Dermoergasilus coleus*, prefer the gill filament for their infestation. On the other hand, the tissue feeding, *Caligodes lacinatus* penetrates the tissue lining of the lower beak and *Bomolochus bellones* clings the operculum. The site specific attachment of parasitic crustaceans involved in the simultaneous infestation was also reported in the previous study on *Strongylura leiura* from which *Lernanthropus tylosuri*, *Caligodes lacinatus* and *Bomolochus bellones* were recovered from the gill filament, the tissue lining of the lower beak and the operculum respectively of the host fish (*Strongylura leiura*) (Aneesh et al. 2013b), suggesting that the site of attachment of parasitic copepods is highly specific even though their hosts are different.

In the present study, all the collected parasitic copepods belonging to the species, *Caligodes lacinatus* (31) and *Bomolochus bellones* (24) and *Dermoergasilus coleus* (32), were invariably matured females carrying egg sac. No single instance of parasitization was noticed by male members of these copepod species (*Caligodes lacinatus*, *Bomolochus bellones* and *Dermoergasilus coleus*) apparently due to the existence of sex specific parasitization. Like in *Strongylura leiura*, 34 out of 252 members of recovered copepod species of *Lernanthropus tylosuri* were males, clinging the genital segments of their parasitic females, apparently in copulatory position. Reports show that only reproductively mature female copepods are parasitic and the males dye after copulation (Jithendran et al. 2008).

Despite the few reports on double parasitism, our knowledge on the occurrence of simultaneous multiple parasitism involving crustaceans is very poor. The occurrence of double parasitism with a copepod, *Pennella* sp. and a cirriped, *Conchoderma virgatum* was reported from the flying fish, *Cypselurus speculiger* (Daniel and Premkumar 1967). The simultaneous infestation of *Nerocila phaiopleura* and the copepod, *Lernaeenicus sprattae* on the engraulidaen fish *Stolephorus commersonii* was reported from Parangipettai (India) (Rajkumar et al. 2006). Double parasitism with the isopod, *Mothocya plagulophora* and the copepod, *Lernaeenicus hemiramphi* was noticed in *Hemiramphus far* (Gopalakrishnan et al. 2010). Daniel and Rao (1967) reported the parasitization of the flying fish by isopod, copepod and cirriped. Recently from our laboratory, the occurrence of double, triple and quadruple parasitism with an isopod *Mothocya renardi* and three copepods such as *Lernanthropus tylosuri*, *Caligodes lacinatus* and *Bomolochus bellones* was reported in the banded needle fish. In the present study, 46.51% (20 out of 43) members of fish, *Strongylura strongylura* showed the occurrence of double parasitism involving one isopod and four copepods in five different combinations. The degree of the occurrence of the combinations *Cymothoa frontalis* and *Lernanthropus tylosuri* (CL) (30%; 6 out of 20), *Lernanthropus tylosuri* and *Caligodes lacinatus* (LC*l*) (25 %; 5 out of 20) and remaining three combinations, *Cymothoa frontalis* and *Caligodes lacinatus* (CC*l*), *Cymothoa frontalis* and *Bomolochus bellones* (CB) and *Lernanthropus tylosuri* and *Bomolochus bellones* (LB) 15 % each. In three combinations (CL, LC*l* and LB), the copepod, *Lernanthropus tylosuri* was found to be common; similarly the isopod, *Cymothoa frontalis* was the common member in the combinations such as CL, CC*l* and CB, signifying its high rate of infestation on the host. In two double parasitic combinations (LC*l* and LB), all members are copepods.

Apart from previous report from our laboratory, no further information is available on triple parasitism by crustaceans. The present study revealed that 17 members (39.5%) of *Strongylura strongylura* had been under triple parasitism with crustacean species. Out of four combinations (CLC*l*, CLB, CLD and LC*l*D) noticed in the triple parasitism, CLC*l* (*Cymothoa frontalis*, *Lernanthropus tylosuri* and *Caligodes lacinatus*) scored the highest percentage (35.29) (Tables 1 and 3). Interestingly, the existence of quadruple parasitism being simultaneously infested by any of the four species of parasitic crustaceans in two different combinations on the fish, *Strongylura strongylura* was also exposed through the present study. However, its frequency was relatively less (9%) occurring only during the months of August, December and March (Tables 1 and 3; Figs 2B and E). The isopod, *Cymothoa frontalis* and copepods, *Bomolochus bellones* and *Dermoergasilus coleus* are found to be the common members in two combinations.

Interestingly, *Lernanthropus tylosuri* appears as a common parasitic crustacean species infesting *Strongylura strongylura* irrespective of the type of parasitism (single, double, triple and quadruple parasitism) involved.

Parasitic crustaceans have negative impacts on their host fishes; their attachment and feeding activities are responsible for any primary diseases that develop due to parasitization (Bharadhirajan et al. 2013). As previously reported in *Strongylura leiura*, the present study, also helped us to identify severe damages induced to the floor of the buccal cavity, the gill filament, the fleshy part of the lower beak, and the inner side of the operculum of the host (*Strongylura strongylura*). Reports showed that, the infestation by parasitic copepods and isopods induce bacterial and viral diseases in parasitized fishes (Nigrelli 1950, Cusack and Cone 1985, Simudu and Tsummoto 1985, Ravichandran et al. 2001, Ravichandran and Ajithkumar 2008). Recent study showed that, the host (*Stolephorus leptolepis*) tissues infected by *Nerocila depressa* were vigorous with disrupted epidermis, damaged muscle fibers and demised collagen matrix; at the pereopod attachment sites, healthy tissues were absent and infested tissues appear to be deteriorated (Rameshkumar and Ravichandran 2013).

In conclusion, the spot-tail needlefish, *Strongylura strongylura* is a potential host for five parasitic crustacean species which showed site specific attachment, may be for avoiding the inter-parasitic competition for space and food. No single instance of parasitization was noticed by male members of these copepod species signifying female specific parasitization. The frequent occurrence of double and triple parasitism and few instance of quadruple parasitism (at the ratio 5:4:1) by parasitic crustaceans noticed on the fish *Strongylura strongylura* indicate that it is not an accidental incident. The multi infestation observed in the present study probably leads the high levels of secondary infections and more studies on this aspect is highly warranted.
